# Hybrid Underwater Image Enhancement via Dual Transmission Optimization and Transformer-Based Feature Fusion

**DOI:** 10.3390/s26020627

**Published:** 2026-01-16

**Authors:** Ning Hu, Shuai Li, Jindong Tan

**Affiliations:** 1 Department of Mechanical, Aerospace and Biomedical Engineering, University of Tennessee, Knoxville, TN 37916, USA; tan@utk.edu; 2 Department of Environmental Engineering Sciences, University of Florida, Gainesville, FL 32611, USA; shuai.li@ufl.edu

**Keywords:** adaptive color correction, image restoration, transformer network, transmission estimation, Uformer, underwater image enhancement, underwater vision

## Abstract

Due to complex underwater environments characterized by severe scattering, absorption, and color distortion, accurate restoration remains challenging. This paper proposes a hybrid approach combining dual transmission estimation, adaptive ambient light estimation with color correction, and a U-Net Transformer (Uformer) for underwater image enhancement. Our method estimates transmission maps by integrating boundary constraints and local contrast, which effectively address visibility degradation. An adaptive ambient light estimation and color correction strategy are further developed to correct color distortion robustly. Subsequently, a Uformer network enhances the restored image by capturing global and local contextual features effectively. Experiments conducted on publicly available underwater image datasets validate our approach. Performance is quantitatively evaluated using widely adopted non-reference image quality metrics, especially Underwater Image Quality Measure (UIQM) and Underwater Color Image Quality Evaluation (UCIQE). The results demonstrate that our proposed method achieves superior enhancement performance over several state-of-the-art methods.

## 1. Introduction

Underwater imaging is crucial in various civil engineering applications, including inspection and maintenance of underwater infrastructures such as bridges, dams, pipelines, ports, offshore platforms, and submerged foundations. High-quality underwater images significantly enhance engineers’ abilities to detect structural defects, perform accurate condition assessments, and make informed decisions for infrastructure maintenance and rehabilitation [1]. However, underwater images typically suffer from severe quality degradation due to absorption, scattering, and color distortion caused by water. Specifically, absorption predominantly reduces the intensity of light, especially in the red spectrum, leading to significant color imbalance, whereas scattering reduces image contrast and obscures structural details critical for infrastructure evaluation [2]. Although traditional restoration methods, such as Dark Channel Prior (DCP) [3] and histogram-based techniques, provide preliminary improvements, they often fail under complex underwater conditions typically encountered in civil engineering inspections due to their rigid assumptions and limited adaptability [4].

Recently, deep learning-based methods have gained significant attention in underwater image enhancement due to their powerful feature extraction capabilities. Methods like WaterNet [5] and Underwater Convolutional Neural Network (UWCNN) [6] demonstrated considerable improvements by learning hierarchical features directly from large datasets. More recently, transformer-based models, particularly the Uformer, have shown promising results due to their capability to capture both global and local context information effectively [7]. Despite these advancements, deep learning methods alone often lack sufficient interpretability and robustness to diverse underwater conditions, emphasizing the need to integrate physically informed models with deep learning frameworks.

The contributions of this paper can be summarized as follows:

(1) We propose a hybrid underwater image enhancement framework that introduces a transmission-guided attention mechanism, in which physically estimated transmission priors are explicitly embedded into the Transformer self-attention process. This design establishes a principled integration paradigm that tightly couples physics-based modeling with Transformer-based global reasoning, rather than treating physical priors as pre- or post-processing components.
(2) Dual Transmission Optimization: A physically informed dual transmission estimation method that integrates boundary constraints and local contrast priors is developed to robustly compensate for wavelength-dependent light attenuation effects and accurately restore degraded scene radiance.
(3) Adaptive Ambient Light Estimation and Color Correction: An effective color correction method is developed to remove severe color casts in underwater images, and the “Shades of Gray” algorithm is subsequently employed to estimate the ambient light color from the color-corrected images, significantly enhancing color restoration accuracy and robustness under varying and complex underwater lighting conditions.
(4) Experimental Validation: A comprehensive evaluation conducted on diverse underwater image datasets is conducted to demonstrate superior visual enhancement performance compared to the state-of-the-art methods.

The remainder of the paper is structured as follows: Section 2 discusses related work on underwater image enhancement methods. Section 3 describes the proposed method in detail, including transmission estimation, adaptive ambient light estimation, and Uformer-based feature enhancement. Experimental setups and evaluation results are presented in Section 4, followed by a detailed ablation study in Section 5. Finally, Section 6 concludes the paper and outlines future research directions.

## 2. Related Work

Traditional underwater image enhancement methods generally focus on adjusting pixel intensity distributions or applying physical priors to improve visibility. Classic methods such as Histogram Equalization (HE) [8], Contrast Stretching [9], and Gamma Correction [10] globally adjust intensity to enhance visibility but often lead to artifacts like noise amplification and unnatural color shifts. To overcome global HE limitations, Adaptive Histogram Equalization (AHE) and Contrast-Limited Adaptive Histogram Equalization (CLAHE) [11] enhance local contrast while mitigating global noise amplification. Retinex-based methods [12] attempt to separate illumination and reflectance components, providing improved brightness and color balance but introduce complexity in parameter tuning and potential artifacts like halos. Physics-based methods, notably the Dark Channel Prior (DCP) [13] and its underwater adaptations such as Wavelength Compensation and Dehazing (WCID) [14], effectively reduce haze and restore color fidelity by modeling underwater optical characteristics. Fusion-based methods [4] merge multiple enhanced outputs to leverage complementary strengths of individual methods, achieving balanced improvements but at higher computational costs.

Deep learning-based methods have recently dominated underwater enhancement due to their adaptive feature extraction capabilities. Early CNN-based architectures, such as MFDNet [15] and WaterNet [5], demonstrated improved color restoration and haze removal by learning from paired datasets. Generative Adversarial Networks (GANs), including FUnIE-GAN [16] and WaterGAN [17], excel in producing visually appealing, high-contrast images and addressing the scarcity of paired datasets by simulating realistic underwater conditions. Hybrid models integrating physical priors with deep learning, like UColor [18] and UIE-Net [12], combine interpretability of traditional methods and adaptive learning of deep networks. Attention mechanisms and Transformers further advance underwater enhancement by dynamically prioritizing important features. Recent Transformer-based methods, such as the U-shaped Transformer proposed by Peng et al. [19], demonstrate superior performance in removing color casts and improving contrast by effectively capturing global contextual dependencies through self-attention.

Transformers, originally developed in natural language processing, have demonstrated strong performance in vision tasks such as image classification, object detection, and semantic segmentation due to their powerful global attention capabilities [20,21,22]. In image restoration and enhancement, models such as IPT [23] and SwinIR [24] exploit attention mechanisms to capture global relationships and fine-grained details effectively. Uformer [7], a U-shaped Transformer for general image restoration, utilizes locally enhanced window-based attention and multi-scale feature modulators to balance global context and local detail efficiently. Despite their computational complexity and data-intensive training requirements, Transformer-based models such as Uformer have shown remarkable potential for underwater image enhancement when provided with sufficient training data and appropriate model constraints.

Recent studies have further explored hybrid architectures that combine convolutional neural networks and Transformer models for underwater image enhancement. In particular, Wang et al. [25] proposed a dual-branch framework that integrates CNN-based representations with Transformer features to improve feature fusion and enhancement performance. While such approaches effectively exploit complementary feature representations from parallel branches, they primarily operate at the feature-fusion level and remain purely data-driven. (As is shown in Table 1).

In contrast, the proposed method differs fundamentally in its design philosophy. Instead of fusing parallel network branches, this work explicitly incorporates physically estimated transmission maps as guidance within the Transformer self-attention mechanism. By allowing physical priors to directly modulate global attention weights, the proposed transmission-guided Uformer establishes a principled integration of physics-based modeling and Transformer-based global reasoning, thereby enhancing robustness and interpretability under complex underwater imaging conditions.

## 3. Methodology

To address the challenges posed by underwater environments, including severe scattering, color distortion, and motion blur, this thesis proposes an improved underwater image enhancement method that integrates dual transmission estimation, adaptive ambient light estimation with color correction, and a Transformer-based multi-scale feature enhancement network (i.e., the Uformer model). The proposed framework includes three main modules: a dual transmission estimation module for image restoration, an adaptive ambient light estimation and color correction module, and a Uformer-based multi-scale Transformer feature enhancement module.

**Figure 1:**
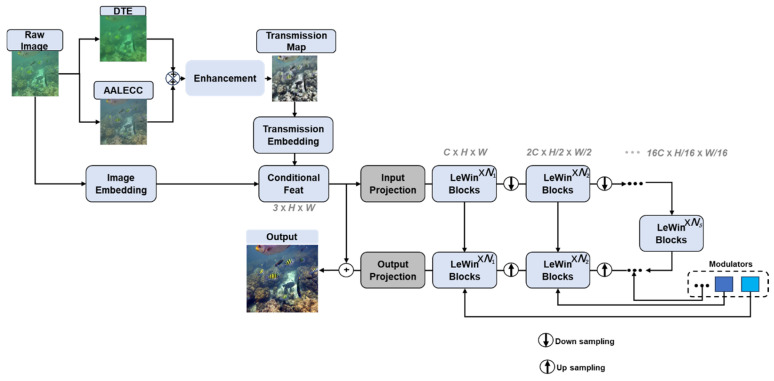
Overall framework.

The overall structure of the proposed underwater image enhancement framework is presented in Figure 1, where it can be seen that the dual transmission estimation module integrates boundary-constraint and local contrast methods to produce a robust transmission map through adaptive Gamma fusion. The adaptive ambient light estimation and color correction module rectifies color distortion effectively, and the Uformer network explicitly incorporates the fused transmission map into the Transformer’s attention mechanism, enhancing underwater images and making them suitable for visual SLAM applications.

### 3.1. Dual Transmission Estimation for Image Restoration

In underwater image formation, image degradation is mainly attributed to scattering effects introduced by the water medium. Among these effects, backward scattering plays a dominant role in reducing image sharpness and contrast, whereas forward scattering generally has a relatively limited influence on the overall imaging quality.

When the bounds of the scene radiance are available, it becomes possible to estimate the feasible range of the transmission. Following this principle, the boundary-constrained transmission can be derived based on Equations (2) and (3), which provide the foundation for estimating transmission under physically meaningful constraints.

(1) $$0\leq t_{b}^{c}(x)\leq t(x)\leq 1,c\in \{r,g,b\}$$

where $t_{b}^{c}(x)$ denotes the lower bound of the transmission, defined as:

(2) $$t_{b}^{c}(x)=\min\left\{\underset{c\in \{r,g,b\}}{\max}\left(\frac{A^{c}-I^{c}(x)}{A^{c}-C_{0}^{c}},\frac{A^{c}-I^{c}(x)}{A^{c}-C_{1}^{c}}\right),1\right\}$$

where $I^{c}(x)$, $A^{c}$, $C_{0}^{c}$, and $C_{1}^{c}$ denote the r, g, and b color channels of I, A, $C_{0}$, and $C_{1}$, respectively.

In the image transmission process, the transmission value within a local image region is assumed to vary smoothly. Therefore, it is reasonable to approximate the transmission as locally constant within a small neighborhood Ω(x). Under this assumption, the transmission for each color channel can be expressed as

(3) $${\tilde{t}}^{c}(x)=\underset{y\in \Omega (x)}{\min}\underset{z\in \Omega (y)}{\max}t_{b}^{c}(z),c\in \{r,g,b\}$$

This local-constancy assumption provides a coarse but effective estimate of the transmission map.

Estimating transmission often requires prior information to obtain an initial approximation. The dark channel prior (DCP) assumes that at least one color channel in a local patch exhibits very low intensity in haze-free images, which motivates selecting the minimum value across color channels within local regions. While DCP has demonstrated effectiveness for atmospheric haze removal, its underlying assumptions do not strictly hold in underwater environments due to wavelength-dependent attenuation. In this work, the DCP principle is referenced only as a conceptual motivation, and the proposed method adapts local contrast and boundary constraints to derive a more suitable transmission estimate for underwater imaging.

In practice, obtaining a reliable transmission estimate typically requires the introduction of prior knowledge to constrain the solution space. A representative example is the dark channel prior (DCP), which is motivated by the observation that haze-free images often contain local regions where at least one color channel exhibits very low intensity. Based on this observation, DCP estimates transmission by selecting the minimum value across color channels within local image patches. While this strategy has proven effective for atmospheric haze removal, its underlying assumptions are frequently violated in underwater environments due to wavelength-dependent absorption and color attenuation.

To address these limitations, alternative physically motivated transmission estimation strategies have been proposed. In particular, boundary-constrained transmission estimation leverages general constraints on scene radiance and reflected light to derive a feasible transmission range without relying on strict channel-based assumptions. This approach has demonstrated improved robustness under challenging imaging conditions. Following this principle, the present study adopts a boundary-constrained formulation to obtain an initial transmission estimate suitable for underwater image restoration.

The core idea of boundary-constrained transmission estimation is to determine the admissible range of transmission based on the reflected light from the scene surface, and then recover the final transmission by assuming spatially smooth behavior within local regions. Accordingly, based on Equation (4), the transmission t(x) can be formulated as follows:

(4) $$t(x)=\frac{\left||I(x)-A|\right|}{\left||J(x)-A|\right|}$$

To avoid numerical instability when the restored radiance **J**(x) approaches the ambient light A, the transmission t(x) is lower-bounded by a small constant ε in implementation, i.e., t(x) = max(t(x), ε), where ε = 0.1 in all experiments.

For a single image, the boundary of the scene surface reflectance J(x) is determined by:

(5) $$C_{0}\leq J(x)\leq C_{1},\forall x\in \Omega$$

where $C_{0}$ and $C_{1}$ denote constants related to the image, and they represent the boundary values of the scene surface reflectance.

**Figure 2:**
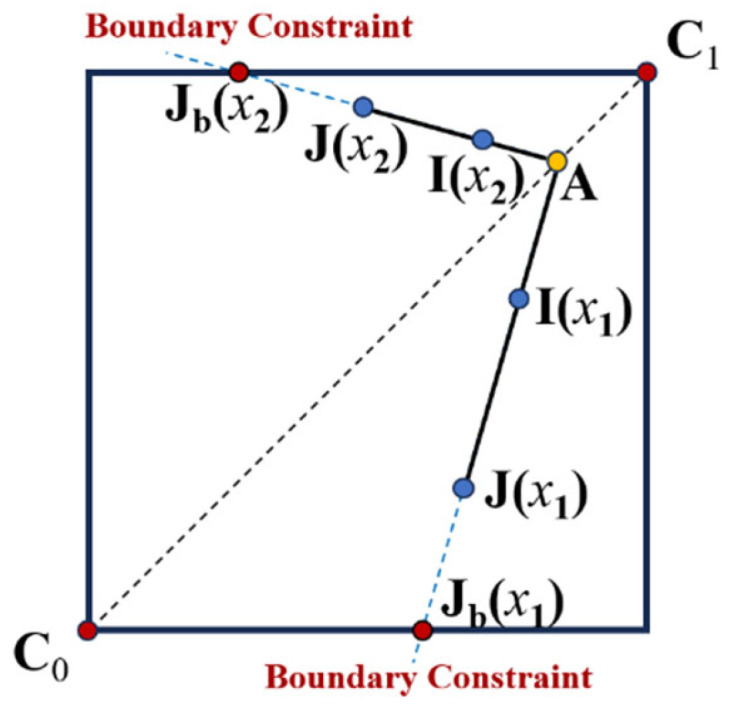
Illustration of the transmission process based on the boundary constraints.

As shown in Figure 2, a scene radiance J(x) must lie within the radiance cube defined by $C_{0}$ and $C_{1}$.

### 3.2. Transmission Map Based on Local Contrast

In dark and turbid underwater environments, imaging systems must overcome various obstacles, including suspended particles, organic materials, and debris, which can blur the imaging process before successfully capturing a scene. Normally, such conditions cause a high amount of particulate matter and organic debris in the water, which makes the medium extremely turbid and murky, blurring the overall image. When the light coming from a scene passes through a polluted aqueous medium, the reflected light is absorbed and scattered by the suspended particulate matter. In highly turbid underwater environments, image degradation is primarily caused by the interaction between light and the water medium, where suspended particles and organic matter introduce significant absorption and scattering effects. These interactions distort the propagation of reflected light from the scene, resulting in reduced visibility, attenuated color information, and blurred structural details in the captured images.

As the level of water turbidity increases, the cumulative absorption and scattering effects become more pronounced, leading to a progressive loss of contrast in local image regions. This observation suggests that local contrast variations can serve as an informative cue for characterizing the degree of transmittance loss induced by water contamination. Motivated by this insight, we estimate a transmission map based on local contrast to describe spatially varying attenuation effects in underwater imagery.

Specifically, for locally contaminated image regions, increased absorption and scattering tend to suppress intensity differences between neighboring pixels, thereby reducing local contrast. Based on this behavior, we assume that the degree of light absorption by the water medium is positively correlated with local contrast degradation within small image neighborhoods. Under this assumption, a local-contrast-based transmission estimation strategy is formulated.

Among various contrast definitions, the Michelson contrast is adopted in this work due to its simplicity and effectiveness in capturing intensity variations, and is defined as follows:

(6) $$\mathrm{Michelson}\;\mathrm{Contrast}=\frac{L_{\max}-L_{\min}}{L_{\max}+L_{\min}}$$

where $L_{\max}$ and $L_{\min}$ are the maximum and minimum pixel values in an image, respectively.

The above definition denotes the overall contrast of an image. To characterize spatial contrast variations, the contrast is computed within a local region centered at x. This local contrast at x is defined as:

(7) $$t_{\beta }(x)=\frac{\underset{y\in \Omega (x)}{\max}I^{gray}(y)-\underset{y\in \Omega (x)}{\min}I^{gray}(y)}{\underset{y\in \Omega (x)}{\max}I^{gray}(y)+\underset{y\in \Omega (x)}{\min}I^{gray}(y)}$$

The local contrast is computed within a fixed square window of size 15 × 15. The grayscale image is obtained using standard luminance conversion from the RGB image, and the resulting local contrast serves as an empirical indicator of turbidity-induced attenuation.

Where Ω(x) denotes the local image block centered at x, and $I^{gray}$ is the grayscale image corresponding to an image *I*.

### 3.3. Transmission Fusion

In this work, two transmission rates ${\tilde{t}}^{c}(x)$ and $t_{\beta }(x)$ are obtained using the boundary constraints and local image contrast, respectively. Then, these two transmission rates are merged to construct the final transmission map. To fully use the information from both transmission rates, this study proposes a simple but effective fusion method for transmission rates. Assume that $t^{c}(x)$ denote the fused transmission rate; then, it can be written that:

(8) $$t^{c}(x)=\max\left({\tilde{t}}^{c}(x),t_{\beta }(x)\right)\times \gamma_{1},c\in \{r,g,b\}$$

where $\gamma_{1}\in (0,1)$ denote the gamma transformation coefficients corresponding to the transmission maps estimated from boundary constraints and local image contrast, respectively.

The final transmission rate $t^{c}(x)$ is computed as a product of the Gamma-transformed versions of ${\tilde{t}}^{c}(x)$ and $t_{\beta }(x)$. Coefficients $\gamma_{1}$ adjust the contributions of the two transmission rates to the final transmission rate, ensuring that $t^{c}(x)$ is neither overestimated nor underestimated after fusion.

The above-defined transmission rate integrates the results of the two transmission estimates. However, since both transmission maps are derived from coarse local estimates based on boundary constraints and local image contrast, where pixels within the same local region share identical transmission values, block artifacts may arise. These artifacts can further lead to halo effects, particularly near regions with sharp intensity transitions such as image edges.

To mitigate these artifacts, it is necessary to refine the coarse transmission estimates. Several approaches have been proposed in the literature, including soft matting and context regularization with weighted L1 norms. In this work, guided filtering is adopted due to its computational efficiency and its ability to preserve edge structures while smoothing homogeneous regions. Specifically, guided filtering is applied with a window radius of 15 pixels and a regularization parameter of 10^−3^, resulting in a smoother and more natural transmission map without introducing halo artifacts.

### 3.4. Adaptive Ambient Light Estimation and Color Correction

In Equation (4), the ambient light A is treated as a known parameter. In practical underwater imaging scenarios, however, ambient light is unknown and must be inferred from the observed image. Existing approaches estimate ambient light by exploiting statistical properties of image intensities. For example, some methods identify candidate ambient-light pixels from regions with high intensity in the dark channel, while others partition the image into subregions and select areas with the highest average brightness as estimates of the ambient illumination.

Although these strategies can provide reliable ambient-light estimation in natural foggy scenes or relatively clear underwater conditions, their performance degrades significantly in turbid waters with severe color bias and spatially varying illumination. Under such conditions, the assumptions underlying global brightness or dark-channel-based selection are frequently violated, leading to inaccurate ambient-light estimation.

To address these challenges, this study adopts an adaptive ambient-light estimation and color correction strategy. Specifically, an initial color correction is first applied to mitigate severe color casts and reduce spectral imbalance. Subsequently, the Shades-of-Gray algorithm is employed to estimate the illumination color from the color-corrected underwater image, enabling a more robust and stable ambient-light estimation under complex underwater lighting conditions. But they are still insufficient for turbid waters with severe color bias and complex lighting. To address this problem, this study employs an adaptive ambient light estimation and color correction method for ambient light estimation, and use the Shades of Gray algorithm to estimate the light source color of the color-corrected underwater images.

Background light $A^{c}$ is estimated using an adaptive approach based on the dark channel prior as follows:

(9) $$A^{c}=\frac{1}{N}\sum_{i=1}^{N}\max(I(i))$$

where $A^{c}$ is the estimated ambient light, and I(i) indicates the brightest pixels selected from the dark channel of an image.

### 3.5. Transformer-Based Feature Enhancement (Uformer)

To further improve the restoration of image details and structural information, this study employs a Transformer-based multi-scale feature enhancement network, namely the Uformer model. It should be noted that although the term “U-Net Transformer” has also been used in prior literature for medical image segmentation tasks (e.g., Petit et al., 2021 [26]), the Uformer adopted in this work follows the general image restoration paradigm proposed by Wang et al., which is fundamentally different in task objective, network architecture, and attention formulation. Unlike traditional Uformer-based restoration approaches that rely solely on data-driven attention mechanisms, the proposed method explicitly incorporates the dual transmission estimation results as physically informed guidance features into the Transformer’s self-attention mechanism. This transmission-guided design enables the network to better exploit global contextual relationships while preserving physically meaningful priors, making it particularly suitable for underwater image enhancement under complex scattering and attenuation conditions.

First, the final transmission map, denoted by $t^{c}(x)$, is projected into a feature embedding through a convolutional layer, which can be expressed as

(10) $$T_{e}(x)=\mathrm{Conv}(t^{c}(x))$$

This projection maps the transmission information into the same embedding space as the input image features.

The embedding $T_{e}\left(x\right)$ is then explicitly introduced into the self-attention mechanism of the Transformer model as a guidance feature, modifying the attention weights as

(11) $$\mathrm{Attention}(Q,K,V,T_{e})=\mathrm{softmax}\left(\frac{QK^{T}}{\sqrt{d}}+\alpha \cdot T_{e}\right)V$$

Here, Q,K and V denote the query, key, and value matrices, respectively. The guidance term $T_{e}\left(x\right)$ is injected via element-wise addition rather than feature concatenation, ensuring dimensional consistency and minimal computational overhead. The weighting parameter α is implemented as a learnable scalar initialized to 1.0, which allows the network to adaptively balance the influence of transmission guidance during training.

It is important to emphasize that the proposed transmission-guided design is fundamentally different from treating transmission estimation as a pre-processing step or performing naive feature concatenation. In conventional pipelines, physical priors are typically applied before a deep network or fused at the feature level, which largely restricts their influence to local appearance adjustment. In contrast, by injecting transmission information directly into the self-attention formulation as shown in Equation (11), the proposed approach allows physically meaningful attenuation cues to modulate global context aggregation. This attention-level integration enables the Transformer to dynamically emphasize regions affected by severe scattering and attenuation, rather than merely refining a pre-restored image.

By integrating physically estimated transmission information at the attention-modulation level, the proposed approach enables the Transformer model to focus more effectively on regions with severe attenuation and scattering in turbid underwater environments, thereby improving robustness and enhancement quality.

### 3.6. Loss Function Design

To ensure robust and stable network training, this study formulates a combined optimization objective consisting of a pixel-level loss (L1 Loss), a structural similarity loss (SSIM Loss), and a perceptual loss, which can be expressed as follows:

(12) $$L_{total}=L_{L1}+\lambda_{SSIM}L_{SSIM}+\lambda_{P}L_{Perceptual}$$

Although both the SSIM loss and the perceptual loss are related to human visual perception, they play complementary roles in the optimization objective. The SSIM loss primarily enforces local structural consistency and edge preservation by measuring luminance, contrast, and structural similarity at a local scale. In contrast, the perceptual loss constrains high-level semantic and textural similarity in the deep feature space, which cannot be adequately captured by pixel-wise or structural metrics alone.

By jointly optimizing these two terms, the network is encouraged to preserve fine structural details while avoiding over-smoothing or unnatural artifacts, leading to more perceptually coherent restoration results.

In this work, the perceptual loss is computed as the L1 distance between feature maps extracted from the relu3_3 and relu4_3 layers of a pretrained VGG-19 network, following common practice in image restoration literature.

Here,

L1 Loss ($L_{L1}$): Measures pixel-wise absolute error, rapidly supervising the network output to approach ground-truth images.

SSIM Loss ($L_{SSIM}$): Supervises accurate recovery of image structures and edges by enforcing structural similarity:

(13) $$L_{SSIM}=1-\mathrm{SSIM}(I_{enhanced},I_{reference})$$

Perceptual Loss ($L_{Perceptual}$): Employs high-level visual features extracted from a pretrained VGG network to supervise the perceptual similarity, significantly enhancing the visual realism of restored images.

Hyperparameters $\lambda_{SSIM}$ and $\lambda_{P}$ are used to balance the contributions of structural and perceptual losses, respectively, ensuring an optimal balance between the pixel fidelity and perceptual image quality.

In summary, the proposed method effectively combines explicit physical-model-based transmission estimation with advanced Transformer-based feature learning, thus significantly enhancing underwater images and offering a robust foundation for downstream visual SLAM tasks.

## 4. Experiments

### 4.1. Experimental Dataset

To validate the effectiveness of the proposed hybrid underwater image enhancement framework, which combines dual transmission optimization and transformer-based feature fusion, under complex underwater conditions, this study conducted a series of systematic experiments using unified experimental settings. Publicly available datasets were selected for validation to ensure the completeness, reproducibility, and credibility of the experimental results. In particular, to assess the performance of the proposed method in real-world underwater scenarios, additional evaluations were conducted on two widely recognized benchmark datasets, namely the EUVP dataset [16] and the underwater image enhancement benchmark dataset (UIEBD) [27]. These two datasets were selected for evaluation because they encompass a broad range of underwater environments across different geographic regions and various water types, thus providing diverse and authentic underwater scenes that can significantly enhance the realism and objectivity of the evaluation process. In addition, additional tests were performed using 330 validation images from the EUVP dataset and 60 challenge images from the UIEBD. However, it should be noted that these two datasets do not include ground truth, which facilitates qualitative and subjective evaluations.

#### Training and Implementation Details

The proposed network was trained in a supervised manner using the UIEB dataset, which contains 890 paired underwater images and corresponding ground-truth reference images. This dataset was exclusively used for training. During training, each input sample consists of a raw underwater image together with its estimated transmission map, while the corresponding ground-truth image from UIEB serves as the supervision signal.

All training images were resized to a spatial resolution of 256 × 256. The network was optimized using the Adam optimizer with an initial learning rate of 1 × 10^−4^ and a batch size of 8, and trained for 300 epochs. The overall loss function consisted of an L1 loss and an SSIM loss, which jointly enforce pixel-level fidelity and structural consistency.

Datasets without ground-truth references, including the UIEBD Challenge-60 subset and the EUVP dataset, were not used for supervised training. Instead, they were employed exclusively as test sets to evaluate the generalization performance of the proposed method on real-world underwater images.

For learning-based comparison methods such as Uformer, Phaseformer, Ushape, and UIECL, we followed the experimental protocols reported in the original papers and employed publicly available pretrained models whenever possible. All methods were evaluated using identical test images, input resolutions, and evaluation metrics to ensure a fair and consistent comparison.

### 4.2. Evaluation Metrics

To comprehensively and objectively evaluate the performance of the proposed hybrid underwater image enhancement method, this study adopted a combination of subjective and objective assessment approaches. Particularly, four widely accepted objective image quality evaluation metrics were selected: UIQM [28], UCIQE [29], NIQE [30] and BRISQUE [31]. These metrics quantitatively measured image fidelity, structural preservation, and perceptual quality from different perspectives.

Furthermore, a subjective visual assessment was conducted to intuitively demonstrate the perceptual effectiveness of the enhanced images obtained by the proposed method, complementing the objective metrics with human visual judgments.

### 4.3. Quantitative Evaluation

To quantitatively validate the performance advantages of the proposed hybrid underwater image enhancement method, a unified evaluation protocol was adopted. Four widely used objective image quality metrics were employed to conduct a comprehensive and systematic performance assessment of the proposed approach.

### 4.4. Objective Evaluation of Datasets

This study also conducted comparative experiments with several state-of-the-art underwater image enhancement approaches, including adaptive gamma correction with weighted distribution (AGCWD), weighted guided image filtering (WGIF), multi-scale retinex with color recovery (MSRCR), U-Shape, UIECL [32] and Phaseformer [33] methods, and the original Uformer model. All methods were implemented and tested under identical computational conditions on a workstation equipped with an Intel^®^ Core™ i7-10700 CPU running at 2.90 GHz and 16 GB of RAM, ensuring a fair comparison across different algorithms.

Quantitative evaluation results in terms of the UIEB and EUVP underwater images are presented in Table 2 and Table 3, respectively.

To quantitatively validate the effectiveness of the proposed method, extensive comparative experiments were conducted on two widely recognized underwater datasets: the UIEBD Challenge-60 and the EUVP330 dataset. Four widely used non-reference image quality metrics, namely UIQM, UCIQE, NIQE, and BRISQUE, were employed to comprehensively assess the performance of the proposed approach against state-of-the-art underwater enhancement methods, including AGCWD, MSRCR, WGIF, Phaseformer, Ushape, and UIECL.

As shown in Table 1 and Table 2, the proposed method achieves the best performance across all four metrics on both underwater datasets, demonstrating clear and comprehensive superiority over existing approaches. In particular, our framework yields the highest UIQM and UCIQE values on UIEBD Challenge-60 and EUVP330, indicating that it produces images with significantly better colorfulness, contrast, sharpness, and overall perceptual fidelity. These improvements highlight the effectiveness of combining dual transmission fusion with adaptive color correction in restoring realistic underwater color and luminance distributions.

Moreover, the proposed method also obtains the lowest NIQE and BRISQUE scores on both datasets, outperforming all competing algorithms in terms of naturalness, structural consistency, and the absence of visual artifacts. This result demonstrates that the integrated Transformer-based feature enhancement not only improves visibility but also preserves natural image statistics more effectively than traditional and deep learning-based techniques.

Overall, the consistent top performance across all four non-reference metrics confirms that the proposed hybrid enhancement framework provides the most balanced and robust improvement in underwater image quality, achieving superior color restoration, contrast enhancement, structural clarity, and perceptual naturalness. These quantitative results clearly validate the practicality and generalization capability of the proposed method in diverse and challenging underwater environments.

### 4.5. Ablation Study

The ablation study is designed not only to evaluate the contribution of individual components, but more importantly to validate different integration strategies between physical priors and Transformer-based enhancement. Rather than simply comparing modules, each ablation variant corresponds to a specific design choice regarding how transmission information is utilized in the overall framework. To further analyze and verify the contribution and effectiveness of each module of the proposed framework, this study conducted comprehensive ablation experiments.

Specifically, the ablation variants are organized to reflect different integration hypotheses: a purely data-driven Transformer without physical guidance, a physical-model-based restoration without deep enhancement, a pre-processing-based integration where physical priors are applied before the Transformer, and the proposed attention-level transmission-guided integration.

Particularly, the following variants of the proposed framework were evaluated:

Only Transmission: This variant used only the dual transmission estimation module without the adaptive color correction module and the Uformer feature enhancement module;

Only Uformer: This variant included only the Uformer network used for image enhancement, without the transmission and adaptive color correction modules;

Transmission + Color Correction (No Uformer): This variant performed transmission estimation combined with adaptive color correction, excluding the Transformer-based enhancement;

Proposed Method (Full model): This model integrated dual transmission estimation, adaptive color correction, and Uformer feature enhancement.

The quantitative results in terms of the UIQM and UCIQE metrics are presented in Table 4 and Table 5. As shown in these tables, the full proposed framework consistently achieves the highest scores across both datasets, demonstrating the strong complementary contributions of each component. The “Trans only” variant yields the lowest UIQM and UCIQE values, indicating that transmission estimation alone cannot sufficiently improve underwater perceptual quality when severe color attenuation and scattering are present. Incorporating adaptive color correction (“Trans + ad”) substantially improves both metrics, confirming that accurate ambient-light estimation plays a crucial role in correcting wavelength-dependent color distortion and recovering global color balance.

The “Uformer-only” configuration performs better than the transmission-only model, as the Transformer enhances structural details and local contrast. However, without the physically informed priors provided by transmission estimation and ambient-light correction, it fails to achieve satisfactory color fidelity or luminance consistency. In contrast, the full proposed framework, which integrates dual transmission fusion, adaptive color correction, and Transformer-based multi-scale feature enhancement, achieves the best performance on both UIEBD Challenge-60 and EUVP330 (UIQM: 4.68/4.64, UCIQE: 0.61/0.61). These improvements indicate that the simultaneous use of physical-model-guided restoration and attention-based feature refinement is essential for producing visually coherent, color-accurate, and perceptually superior underwater images.

Furthermore, UIQM and UCIQE were intentionally chosen as the primary metrics for ablation analysis because they were specifically developed for underwater optical imaging, directly reflecting changes in color fidelity, contrast enhancement, and brightness uniformity. In contrast, naturalness-based metrics (NIQE and BRISQUE) depend on terrestrial natural-image statistics and therefore do not reliably capture component-level effects in underwater enhancement pipelines. Using UIQM and UCIQE ensures that the ablation study accurately reflects the impact of each module under true underwater imaging conditions. Overall, the ablation results verify that each component contributes uniquely to the final enhancement quality and that their integration is crucial for achieving state-of-the-art performance.

### 4.6. Subjective Evaluation of Datasets

To further evaluate the perceptual effectiveness of the proposed method, subjective visual comparisons were performed on representative images selected from two challenging benchmark datasets: the UIEBD Challenge-60 and EUVP330. These images illustrate various typical underwater degradations, including severe color distortion, significant haze, low contrast, and poor visibility. The results clearly demonstrate that our approach consistently achieves superior visual quality compared to state-of-the-art methods. Specifically, the proposed method effectively removes color casts, significantly suppresses haze, restores accurate colors, and enhances structural clarity, thereby providing visually realistic and perceptually pleasing underwater images.

**Figure 3:**
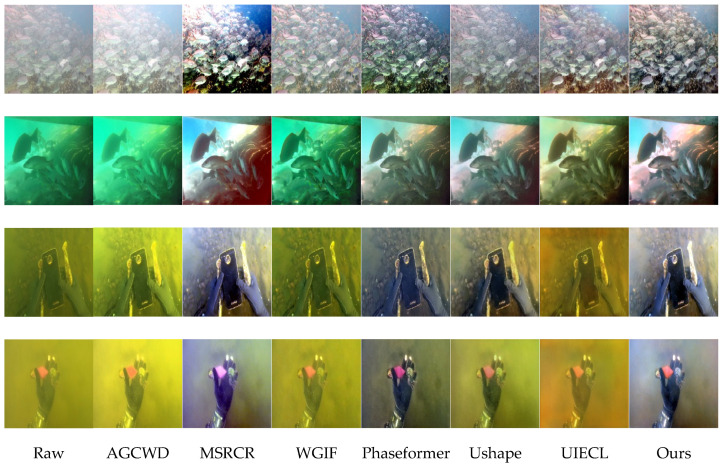
Visual comparison results from challenge-60.

Figure 3 presents visual enhancement results from the UIEBD Challenge-60 dataset, illustrating representative underwater images with distinctive degradation characteristics such as severe haze, significant color casts, and low contrast. Compared with state-of-the-art methods (AGCWD, MSRCR, WGIF, Phaseformer, Ushape, and UIECL), our proposed method consistently produces more visually appealing images with better clarity, naturalness, and accurate color restoration.

Row 1 (fish shoal): The original image is heavily degraded by haze, resulting in poor visibility and washed-out colors. AGCWD and WGIF methods inadequately address haze removal and produce dull results. MSRCR and Phaseformer over-enhance the image, introducing unnatural brightness and colors. Ushape and UIECL improve visibility, but colors appear slightly pale. In contrast, our method effectively reduces haze, enhances contrast, and restores vibrant yet realistic colors, clearly presenting fish details and underwater textures without introducing unnatural artifacts.

Row 2 (fish near the seabed): The raw image suffers from severe greenish and bluish color distortion. AGCWD and MSRCR result in oversaturated and unnatural color shifts. WGIF and Phaseformer provide limited improvements, leaving significant color casts unresolved. Ushape and UIECL partially correct the color, yet image details remain obscure. Our method provides superior color correction, significantly removing the unnatural greenish tint and enhancing details of fish and seabed structure, resulting in a natural-looking underwater scene.

Row 3 (hands holding a phone underwater): The original image shows strong yellowish-brownish color distortion and haze. AGCWD, WGIF, and MSRCR methods fail to effectively correct these color biases. Phaseformer and Ushape methods partially alleviate color issues but leave residual haze and slight color distortion. Our method effectively corrects the severe color bias, accurately restoring natural colors of both the gloves and phone, and notably enhancing clarity and visual details, providing a significantly more realistic visual perception.

Row 4 (glove holding object underwater): Original severe yellow-green cast significantly obscures the glove and object details. Most comparison methods either fail to correct color cast adequately (AGCWD, WGIF, Phaseformer) or introduce unnatural colors and limited detail improvement (MSRCR, Ushape, UIECL). Our method remarkably corrects color distortion, reduces haze, and recovers clearer structural details, showing natural and realistic colors, thus significantly improving the visual quality.

**Figure 4:**
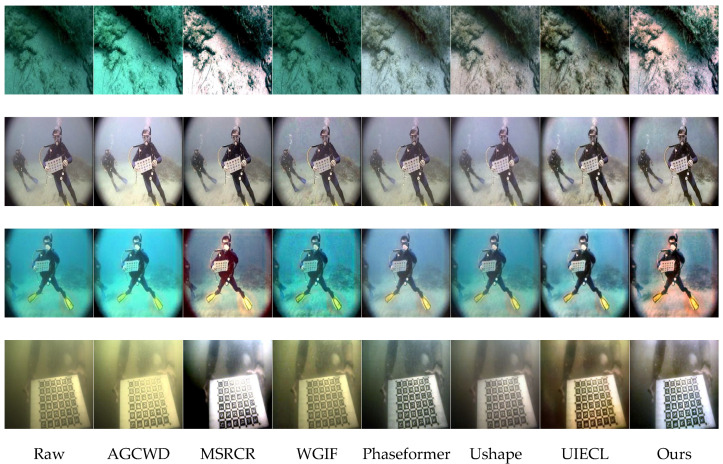
Visual comparison results from EUVP330.

Figure 4 illustrates visual enhancement results selected from the EUVP330 dataset, featuring underwater images characterized by various complex degradation issues such as severe haze, evident color distortion, and poor visibility. Compared with other state-of-the-art approaches (AGCWD, MSRCR, WGIF, Phaseformer, Ushape, and UIECL), our proposed method consistently demonstrates superior visual enhancement, particularly in effectively correcting color bias, restoring realistic appearance, and enhancing structural clarity.

Row 1 (underwater rock and sand texture): The original image shows significant greenish color cast and haze, severely affecting texture recognition. The AGCWD, WGIF, and Phaseformer methods only slightly alleviate haze, leaving noticeable color distortions. MSRCR produces overly bright results, losing realistic texture details. Ushape and UIECL partially improve color accuracy but still retain residual color casts. In contrast, our method remarkably reduces the haze and effectively corrects the color cast, clearly presenting realistic sand and rock textures with natural and visually pleasing colors.

Row 2 (diver holding calibration board with significant vignette): This challenging scenario contains severe vignetting effects and moderate haze, significantly impairing the visibility of the divers and calibration board. The AGCWD, WGIF, MSRCR, and Phaseformer methods inadequately address these issues, leaving the vignette or haze partially unresolved. Ushape and UIECL mitigate the problems but still maintain unnatural brightness or slight residual haze. Our method achieves the most balanced enhancement, effectively alleviating the vignette effect, substantially reducing haze, and restoring a natural and clear underwater scene, thus providing improved clarity and realistic color rendition.

Row 3 (diver with notable bluish-green color distortion): Severe bluish-green tint and hazy conditions obscure crucial details of the diver and surrounding underwater features. AGCWD, WGIF, and Phaseformer fail to adequately correct the severe color distortions. MSRCR introduces unnatural color shifts and artifacts. Ushape and UIECL partially restore colors, though visibility remains somewhat impaired. In contrast, our method successfully corrects the severe color distortions, significantly reduces haze, and visibly restores realistic colors and detailed structures of the diver and the underwater environment.

Row 4 (underwater calibration pattern): The raw image suffers from strong yellowish haze and low contrast, hindering readability and structural detail recognition. AGCWD and WGIF methods only slightly improve the contrast without adequately addressing color distortions. MSRCR and Phaseformer produce artificial colors and unnatural appearance. Ushape and UIECL achieve moderate enhancements but leave residual color distortions. Our proposed method notably removes the yellowish haze, significantly enhances structural clarity, and restores accurate, natural colors, resulting in superior readability and realistic image appearance.

In summary, the subjective evaluations clearly demonstrate that our proposed method consistently delivers visually superior results. Compared to state-of-the-art approaches, our method intuitively excels in effectively correcting severe color distortions, significantly enhancing clarity, suppressing haze, and restoring realistic and natural underwater scenes. Thus, from a perceptual viewpoint, our algorithm proves highly effective and visually appealing.

### 4.7. Computational Efficiency Analysis

As is shown in Table 6, the computational analysis demonstrates that the proposed method introduces only a marginal overhead compared to the baseline Uformer, despite incorporating transmission-guided attention modulation. Benefiting from the lightweight embedding design and the efficient integration of the transmission map into the attention mechanism, the model maintains nearly identical parameters and FLOPs while achieving faster GPU inference speed. In contrast, Phaseformer exhibits significantly higher computational cost, and traditional methods such as AGCWD and WGIF achieve faster runtimes but deliver inferior enhancement quality. Overall, the proposed method achieves an excellent balance between efficiency and visual performance, making it well suited for real-time or near-real-time deployment in underwater robotic perception and field inspection systems.

## 5. Discussion

The proposed hybrid underwater image enhancement framework incorporates both physically grounded restoration and Transformer-based feature refinement, and its effectiveness is supported by comprehensive objective and subjective evaluations. The quantitative results on the UIEBD Challenge-60 and EUVP330 datasets demonstrate that our method achieves superior performance across all major underwater quality metrics, including UIQM, UCIQE, NIQE, and BRISQUE. These metrics collectively indicate that the proposed approach attains the best overall balance of color fidelity, contrast enhancement, structural preservation, and naturalness among the compared state-of-the-art methods.

Beyond quantitative comparisons, extensive subjective evaluations further confirm the perceptual advantages of the proposed method. Representative samples from both datasets display a wide variety of underwater degradations—such as severe color distortion, heavy haze, vignetting, low contrast, and poor visibility—that pose significant challenges to underwater imaging systems. Across all cases, the proposed method consistently produces the most visually realistic, color-accurate, and structurally clear outputs.

In the UIEBD Challenge-60 dataset (Figure 3), scenes involving fish shoals, seabed environments, and objects held underwater demonstrate the limitations of conventional enhancement techniques. Traditional methods such as AGCWD and WGIF inadequately remove haze and often produce muted colors. MSRCR and Phaseformer tend to over-enhance, generating unnatural brightness and color shifts. While Ushape and UIECL offer partial improvements, their outputs often remain pale or insufficiently corrected. In contrast, our method effectively reduces haze, restores vivid yet natural colors, and enhances scene details without introducing artifacts. It successfully corrects strong green, blue, or yellow color casts and recovers fine structural information—such as fish textures, seabed patterns, and object edges—resulting in images that appear more faithful to real underwater environments.

Similarly, on the EUVP330 dataset (Figure 4), which includes scenes with rock textures, divers, and calibration boards, the proposed method consistently produces visually coherent enhancement results. Severe greenish or bluish color biases and haze are effectively corrected, yielding balanced and realistic color tones. Competing methods often exhibit insufficient haze suppression (AGCWD, WGIF), unnatural brightness (MSRCR), or incomplete color correction (Phaseformer, Ushape, UIECL). In challenging scenarios affected by vignetting or strong spectral shifts, the proposed approach demonstrates improved color consistency and local structural clarity. Fine details such as diver outlines, textures, and calibration patterns are better preserved, contributing to enhanced readability and perceptual coherence.

Overall, the convergence of objective metrics and subjective visual assessments indicates that the proposed framework provides a robust and balanced improvement in underwater image quality. The dual transmission fusion mitigates wavelength-dependent attenuation and scattering, the adaptive color correction improves ambient-light estimation, and the Transformer-based multi-scale feature enhancement further refines structural details and contrast. Together, these components contribute to underwater images that are not only quantitatively competitive but also visually coherent and perceptually plausible.

From a practical perspective, the proposed method is well suited for challenging underwater environments and may serve as a strong preprocessing module for downstream vision tasks. From a mechanism perspective, improving underwater image quality directly benefits downstream vision tasks by enhancing feature stability, contrast consistency, and color fidelity. In robotic perception pipelines, tasks such as object detection and recognition rely heavily on reliable low-level features and consistent photometric properties. Visual SLAM, in particular, is highly sensitive to feature stability and photometric consistency. Severe color distortion and scattering-induced haze often lead to degraded feature matching and unstable tracking, while enhanced images with improved contrast and color balance can significantly improve robustness. Prior studies have shown that underwater image enhancement can positively impact downstream tasks such as object detection and navigation by improving feature visibility and reducing perceptual ambiguity [16,17]. Therefore, although not explicitly evaluated in this work, the proposed enhancement framework is expected to provide practical benefits for underwater robotic vision and exploration systems. A systematic evaluation of its impact on applications such as underwater inspection, marine robotics, and visual SLAM will be an important direction for future work.

Despite the strong performance observed in most scenarios, the proposed method has several limitations under extreme underwater conditions. In very low-light environments, the estimated transmission may become less stable, which can lead to amplified noise after enhancement. Under conditions dominated by strong backscatter, transmission estimation may be locally overestimated, occasionally resulting in overcorrection and reduced local contrast. In deep-water environments with severe wavelength-dependent attenuation, residual color shifts may still remain even after adaptive color correction. In addition, highly non-uniform artificial illumination can violate the underlying assumptions of spatially smooth transmission, leading to inconsistent enhancement across the image. These limitations reflect the inherent challenges of underwater optical modeling and indicate directions for further improvement.

## 6. Conclusions

In this paper, we presented a hybrid underwater image enhancement framework that integrates dual transmission optimization, adaptive ambient-light-based color correction, and Transformer-driven multi-scale feature refinement. By combining physically grounded modeling with data-driven feature learning, the proposed method effectively addresses the major challenges of underwater imaging, including severe color degradation, scattering-induced haze, low contrast, and structural smearing.

Extensive evaluations on two widely used benchmark datasets—UIEBD Challenge-60 and EUVP330—demonstrate that the proposed method achieves **state-of-the-art performance across all four major non-reference underwater quality metrics**, namely UIQM, UCIQE, NIQE, and BRISQUE. These quantitative results indicate substantial improvements in color fidelity, contrast enhancement, structural clarity, and statistical naturalness. Complementary subjective visual comparisons further confirm that our approach consistently produces more realistic, visually coherent, and perceptually pleasing underwater images than existing traditional and deep learning–based techniques. The restored outputs exhibit accurate color balance, reduced haze, enhanced detail visibility, and natural appearance across a wide spectrum of underwater conditions.

The strong empirical performance demonstrates the effectiveness of incorporating both physical priors and Transformer-based global contextual reasoning into a unified enhancement framework. Beyond improving visual perception, the enhanced images produced by our method are expected to significantly benefit downstream underwater vision tasks—such as feature detection, pose estimation, inspection analysis, and visual SLAM—where image clarity and color accuracy are critical to algorithmic robustness and operational reliability.

Although the proposed method achieves superior performance, future work will explore further integration with underwater robotic perception systems, including real-time adaptation, model compression for embedded platforms, and tight coupling with SLAM pipelines. Additionally, incorporating multimodal cues such as sonar, depth sensors, or inertial measurements may further improve robustness under extreme turbidity or low-light conditions.

Overall, the results presented in this study establish the proposed framework as a powerful and practical solution for underwater image enhancement, offering strong potential for widespread applications in marine robotics, environmental monitoring, and underwater infrastructure inspection.

## Figures and Tables

**Table 1:** Conceptual comparison of representative underwater image enhancement methods, highlighting differences in physical prior usage and integration level within deep learning and Transformer-based frameworks.

Method	Physical Prior Usage	Integration Level	Transformer Role
UColor	Implicit	Feature embedding	Feature weighting
UIE-Net	Implicit	Preprocessing	CNN enhancement
Phaseformer	None	Attention (phase)	Phase-based attention
U-shaped Transformer	None	Feature hierarchy	Global context modeling
Proposed	Explicit	Attention modulation	Transmission-guided attention

**Table 2:** The objective comparison results of different methods on underwater images from UIEBD Challenge-60. (**Bold** and underline indicate the **best** and second best results).

Method	Ours	AGCWD	MSRCR	WGIF	Phaseformer	Ushape	UIECL
UIQM	**4.68**	2.42	4.64	3.12	3.43	4.13	4.17
UCIQE	**0.61**	0.53	0.61	0.50	0.53	0.54	0.55
NIQE	**3.75**	5.15	5.80	5.12	4.55	4.50	4.88
BRISQUE	**22.91**	30.25	32.08	30.81	38.71	24.57	26.46

**Table 3:** The objective comparison results of different methods on underwater images from EUVP330. (**Bold** and underline indicate the **best** and second best results).

Method	Ours	AGCWD	MSRCR	WGIF	Phaseformer	Ushape	UIECL
UIQM	**4.64**	1.64	4.44	2.54	3.02	3.49	3.71
UCIQE	**0.61**	0.51	0.60	0.50	0.55	0.55	0.57
NIQE	**3.64**	4.49	4.70	4.64	3.91	4.22	4.36
BRISQUE	**27.20**	36.13	35.01	38.25	41.81	31.07	30.97

**Table 4:** The ablation study’s results regarding the UIQM metric.

	Challenge-60	EUVP330
Proposed	**4.68**	**4.64**
Trans + ad	3.07	2.81
Trans only	1.72	1.56
Uformer	2.54	1.95

**Table 5:** The ablation study’s results regarding the UCIQE metric.

	Challenge-60	EUVP330
Proposed	**0.61**	**0.61**
Trans + ad	0.45	0.44
Trans only	0.44	0.43
Uformer	0.47	0.46

**Table 6:** Computational efficiency comparison of different underwater image enhancement methods on an NVIDIA RTX 3080 GPU.

Method	Params (M)	FLOPs (G)	Runtime (ms)
Ours	19.9	62.1	142
Uformer	20.6	62.4	158
Phaseformer	23.1	70.2	181
Ushape	18.4	55.7	149
WGIF	-	-	34
AGCWD	-	-	29

## Data Availability

This study used publicly accessible underwater image datasets. The UIEBD is freely available at https://li-chongyi.github.io/proj_benchmark.html (accessed on 9 February 2020) and the EUVP dataset at https://irvlab.cs.umn.edu/resources/euvp-dataset (accessed on 9 February 2020). No additional data were created or analyzed beyond these existing resources.
